# Cholecystectomy reduces the risk of myocardial and cerebral infarction in patients with gallstone-related infection

**DOI:** 10.1038/s41598-022-20700-y

**Published:** 2022-10-06

**Authors:** Seon Mee Park, Hyun Jung Kim, Tae Uk Kang, Heather Swan, Hyeong Sik Ahn

**Affiliations:** 1 Department of Internal Medicine, Chungbuk National University College of Medicine and Chungbuk National University Hospital, Cheongju, Republic of Korea; 2 Department of Preventive Medicine, Korea University College of Medicine, 126-1, 5-ga, Inchon-ro, Seoul, 136-705 Republic of Korea; 3 Health and Wellness College, Sungshin Women’s University, Seoul, Republic of Korea; 4 Department of Public Health, Graduate School, Korea University, Seoul, Republic of Korea

**Keywords:** Gastroenterology, Risk factors

## Abstract

We compared the risk of myocardial infarction (MI) or cerebral infarction (CI) in patients with or without-gallstone-related infection (GSI) and change in the risk following cholecystectomy. GSI (n = 84,467) and non-GSI (n = 406,800) patients with age- and sex-matched controls (n = 4,912,670) were identified from Korean population based data. The adjusted hazard ratios (aHRs) of MI or CI were analyzed in both groups treated with or without cholecystectomy. Subgroup analysis was performed for both sexes and different ages. The risk of MI or CI was higher in the GSI group than in the non-GSI group (aHR for MI; 1.32 vs. 1.07, aHR for CI; 1.24 vs. 1.06, respectively). The risk reduction rate of MI following cholecystectomy was 11.4% in the GSI group, whereas it was 0% in the non-GSI group. The risk of CI after cholecystectomy was more reduced in the GSI group than in the non-GSI group (16.1% and 4.7%, respectively). The original risk of MI or CI in patients with gallstones and risk reduction rates following cholecystectomy were higher in females and younger patients than in males and older patients. Increased risk of MI or CI and greater risk reduction following cholecystectomy were seen in patients with GSI.

## Introduction

Gallstones are one of the leading cause of hospital admissions, with a prevalence as high as 5–15% in worldwide^1^. The majority of gallstone carriers remain asymptomatic; however, one in five develop biliary pain or gallstone-related complications. Complicated gallstones developed at a rate of 7.2% during 17.5 year follow up^2^. They developed mostly due to gallstone-related infection (GSI) such as acute cholecystitis or acute cholangitis^3^. Cholecystectomy is usually indicated for symptomatic gallstones^4^, and it is widely indicated in laparoscopic era^4^.

An association between gallstones and cardiovascular diseases (CVD) has been reported in several studies^5–7^, demonstrating up to 1.1–2.0-fold CVD risks in patients with gallstones. Shared cardio-metabolic risk factors have been identified as a cause of this association such as smoking, alcohol drinking, physical inactivity, obesity, and health factors including abnormalities in lipid profile, blood pressure, or glucose level^8^. Shared common pathogenesis of CVD and gallstones has been proposed as an another cause of this association. Chronic inflammation and oxidative stress are the main causes of vascular atherosclerosis^9,10^ and gallstone formation^11^.

There is solid evidence that chronic infection in humans plays a role in the development of atherogenesis^12^. Recent studies have emphasized the role of bacterial infection in the development of atherosclerosis^13,14^. Gram-negative bacteria, major pathogens of GSI, produce lipopolysaccharide (LPS) or inflammatory mediators in the serum of patients. Vascular CVD, such as acute coronary syndrome, myocardial infarction (MI), and cerebral infarction (CI)^15^ may increase in patients with GSI compared to non-GSI. However, only few studies have been reported with mixed results^5,16–18^.

Moreover, existing literature shows conflicting findings regarding the role of cholecystectomy in CVD risk. Several studies have demonstrated equivocal CVD risks after cholecystectomy^19–21^, while others have reported increased^20^ or reduced^17^ risks of CVD following cholecystectomy. Cholecystectomy may have a role in eliminating the source of inflammation. Because control of inflammation is considered a promising treatment for CVD^22^, cholecystectomy may reduce the risk of MI or CI in patients with GSI. The apparent increased CVD risk in the cholecystectomy group may not be due to gallbladder removal but by confounding variables such as gallstone-related inflammation. This hypothesis is supported by the finding that CVD risk decreased after cholecystectomy by adjustment of severity of gallstone-related inflammation^5^. However, growing evidences were also reported that cholecystectomy itself induces metabolic syndrome, which is a risk factor for CVD^23^. We suggest that the effects of cholecystectomy on CVD risk may depend on GSI or non-GSI state.

This population-based large cohort study aimed to assess the effects of cholecystectomy on the risk of MI or CI in patients with GSI or non-GSI. This study used the Korean National Health Insurance (KNHI) and Korean National Health Screening Program (KNHSP) databases, which have reliable data regarding lifestyle factors, gallstone diagnoses, and cholecystectomy, to assess the risk of MI or CI with nearly full population coverage. Additional analyses were performed for both sexes and different age groups.

## Results

### Patient characteristics

**Figure 1:**
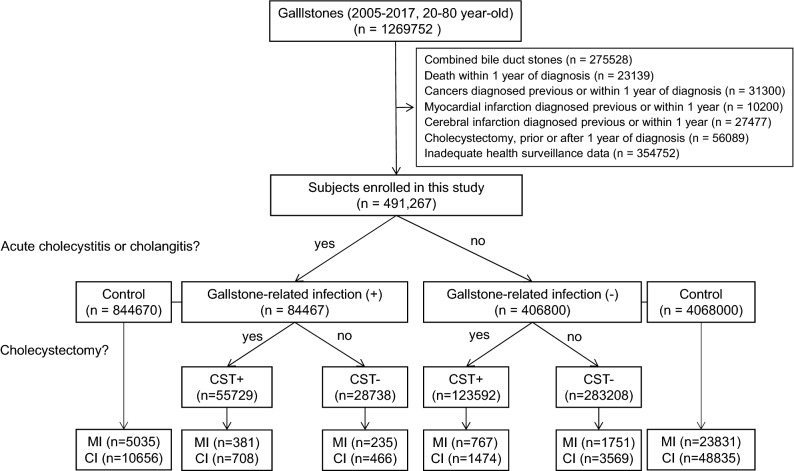
Flow chart of the study. MI, myocardial infarction; CI, cerebral infarction; CST, cholecystectomy.

Gallstone patients (n = 491,267) and controls (n = 4,912,670) are included in this study (Fig. 1) and their demographics are presented in Table 1. The male-to-female ratio was 51.8/48.2 and mean age was 53 years. There were no differences in clinical characteristics between gallstone patients and controls except for BMI; the gallstone group had a higher rate of obesity than the controls (*P* = 0.2). Among gallstone patients, the GSI (n = 84,467, 17.2%) and non-GSI (n = 406,800, 82.8%) groups were identified. The GSI group included patients with acute cholecystitis (n = 80,076, 94.8%) or acute cholangitis (n = 4,391, 5.2%). The GSI and non-GSI groups revealed similar clinical features in terms of age, sex, and other risk factors. However, the cholecystectomy rates were quite different, with 66.0% in the GSI group and 30.4% in the non-GSI group.

**Table 1:** Demographics of study populations with or without gallstone-related infection and controls.

Characteristics	Gallstones (+)						Controls	
	Gallstone-related infection (+)		Gallstone-related infection (-)		Total			
No. of persons	84,467		406,800		491,267		4,912,670	
**Sex**								
Male	44,369	52.5	209,954	51.6	254,323	51.8	2,543,230	51.8
Female	40,098	47.5	196,846	48.4	236,944	48.2	2,369,440	48.2
Ages (years), mean ± SD	54.51	12.59	54.81	13.19	54.61	12.66	54.56	12.69
**Ages stratified, years**								
20–29	2212	2.6	10,163	2.5	12,375	2.5	123,750	2.5
30–39	9503	11.3	41,398	10.2	50,901	10.4	509,010	10.4
40–49	17,917	21.2	88,919	21.9	106,836	21.8	1,068,360	21.8
50–59	22,143	26.2	117,320	28.8	139,463	28.4	1,394,630	28.4
60–69	19,316	22.9	95,141	23.4	114,457	23.3	1,144,570	23.3
70+	13,376	15.8	53,859	13.2	67,235	13.7	672,350	13.7
**Cholecystectomy**								
Yes	55,729	66.0	123,592	30.4	179,321	36.5		
No	28,738	34.0	283,208	69.6	311,946	63.5		
**SBP/DBP (mmHg)**								
< 120/ < 80	28,117	33.3	131,965	32.4	160,082	32.6	1,655,334	33.7
120–129/ < 80	10,017	11.9	49,422	12.1	59,439	12.1	588,377	12.0
130–139/80–89	30,663	36.3	149,700	36.8	180,363	36.7	1,763,149	35.9
140–179/90–119	15,263	18.1	73,840	18.2	89,103	18.1	881,723	18.0
180+/120+	390	0.5	1825	0.4	2215	0.5	23,587	0.5
**Pulse pressure (mmHg)**								
< 40	12,896	15.3	60,838	15.0	73,734	15.0	745,937	15.2
40–59	60,214	71.3	291,799	71.7	352,013	71.7	3,511,413	71.5
≥ 60	11,340	13.4	54,115	13.3	65,455	13.3	654,804	13.3
**Fasting plasma glucose (mg/dl)**								
< 100	51,307	60.7	247,957	61.0	299,264	60.9	3,154,907	64.2
100–125	24,463	29.0	118,045	29.0	142,508	29.0	1,346,085	27.4
≥ 126	8668	10.3	40,682	10.0	49,350	10.0	410,396	8.4
**Cholesterol (mg/dl)**								
< 200	48,468	57.4	230,295	56.6	278,763	56.7	2,737,743	55.7
200–239	25,673	30.4	125,469	30.8	151,142	30.8	1,557,014	31.7
≥ 240	10,287	12.2	50,885	12.5	61,172	12.5	616,144	12.5
**BMI (kg/height, m**^**2**^**)**								
< 18.5	1924	2.3	9390	2.3	11,314	2.3	138,799	2.8
18.5–25.0	45,334	53.7	222,092	54.6	267,426	54.4	3,022,252	61.5
≥ 25.0	37,209	44.1	175,318	43.1	212,527	43.3	1,751,619	35.7
**Smoking (pack/year)**								
None	53,249	63.0	260,221	64.0	313,470	63.8	3,174,589	64.6
1–9	8734	10.3	40,231	9.9	48,965	10.0	490,480	10.0
10–19	8927	10.6	42,272	10.4	51,199	10.4	513,203	10.5
20–29	5809	6.9	28,184	6.9	33,993	6.9	329,341	6.7
30–39	3488	4.1	16,613	4.1	20,101	4.1	190,061	3.9
40+	3422	4.1	15,180	3.7	18,602	3.8	166,347	3.4
**Alcohol drinking**								
None	41,635	49.3	196,675	48.3	238,310	48.5	2,333,815	47.5
< one/month	18,235	21.6	84,683	20.8	102,918	20.9	1,089,546	22.2
< one/week	2352	2.8	12,495	3.1	14,847	3.0	153,866	3.1
≥ one/week	11,947	14.1	59,542	14.6	71,489	14.6	701,408	14.3
**Physical activity**								
None	42,204	50.0	199,106	48.9	241,310	49.1	2,365,622	48.2
1–2/week	23,866	28.3	116,096	28.5	139,962	28.5	1,414,395	28.8
3–4/week	9863	11.7	49,307	12.1	59,170	12.0	603,795	12.3
5–6/week	3857	4.6	19,004	4.7	22,861	4.7	242,165	4.9
Every day	3268	3.9	16,226	4.0	19,494	4.0	201,111	4.1

All data represent number of patients (percent); Standardized mean difference (SD) ≤ 0.10 indicates a negligible difference between the two cohorts. All SD were less than 0.1 except BMI (*P* = 0.2 for gallstone patients vs. matched controls); SBP/DBP, systolic blood pressure/diastolic blood pressure; BMI, body mass index.

### Incidences and risks of MI or CI in GSI or non-GSI groups

The cumulative incidence of MI or CI during the 15-year follow-up (4,403,817 person-years) was observed among gallstone patients without cholecystectomy and controls. The incidence of MI or CI was highest in the GSI group, followed by the non-GSI group and controls. The incidence rate ratios (IRR, 95% confidence intervals [CI]) and adjusted hazard ratio (aHR, 95% CI) of MI and CI are presented (Table 2). Among gallstone patients who did not undergo cholecystectomy, the IRR and aHR of MI were higher in the GSI group (1.40 [1.23–1.60] and 1.32 [1.15–1.50], respectively) than in the non-GSI group (1.10 [1.04–1.15] and 1.07 [1.02–1.12], respectively). The IRR and aHR for CI were also higher in the GSI group (1.32 [1.20–1.44] and 1.24 [1.13–1.36], respectively) than in the non-GSI group (1.09 [1.05–1.13] and 1.06 [1.03–1.10], respectively).

**Table 2:** Crude and adjusted risks of myocardial infarction or cerebral infarction according to gallstone-related infection and cholecystectomy.

	Gallstones								
	All	GSI				Non-GSI			
	aHR (95% CI)	Events/person-time	Rate* (95% CI)	IRR (95% CI)	aHR (95% CI)	Events/person-time	Rate (95% CI)	IRR (95% CI)	aHR (95% CI)
**Myocardial infarction**									
Matched control		5,035/ 5,414,099	9.30 (9.05–9.56)			23,831/26,623,120	8.95 (8.84–9.07)		
Without cholecystectomy	1.10 (1.05–1.15)	235/180,021	13.05 (11.49–14.83)	1.40 (1.23–1.60)	1.32 (1.15–1.50)	1,751/1,785,157	9.81 (9.36–10.28)	1.10 (1.04–1.15)	1.07 (1.02–1.12)
With cholecystectomy	1.10 (1.03–1.16)	381/356,528	10.69 (9.67–11.82)	1.15 (1.03–1.28)	1.17 (1.05–1.29)	767/849,656	9.03 (8.41–9.69)	1.01 (0.94–1.08)	1.07 (1.00–1.15)
Risk reduction by cholecystectomy(%)^#^	0.0 (-0.9–1.9)				11.4 (8.7–14.0)				0.0 (-2.7–2.0)
**Cerebral infarction**									
Matched control		10,656/ 5,392,612	19.76 (19.39–20.14)			48,835/ 26,526,916	18.41 (18.25–18.57)		
Without cholecystectomy	1.08 (1.05–1.12)	466/179,145	26.01 (23.75–28.48)	1.32 (1.20–1.44)	1.24 (1.13–1.36)	3,569/1,778,205	20.07 (19.42–20.74)	1.09 (1.05–1.13)	1.06 (1.03–1.10)
With cholecystectomy	1.02 (0.98–1.06)	708/355,075	19.94 (18.52–21.46)	1.01 (0.93–1.09)	1.04 (0.96–1.12)	1,474/ 846,798	17.41 (16.54–18.32)	0.95 (0.90–1.00)	1.01 (0.96–1.07)
Risk reduction by cholecystectomy(%)	5.6 (5.4–6.7)				16.1 (15.0–17.6)				4.7 (2.7–6.8)

*, No. of events/10^4^ person-time; GSI, gallstone-related infection; MI, myocardial infarction; CI, cerebral infarction; IRR, incidence rate ratio; aHR, adjusted hazard ratio; #, HR (C^–^C^+^)/C^–^ × 100 (%).

### Effects of cholecystectomy on the incidences and risks of MI or CI in both GSI or non-GSI groups

**Figure 2:**
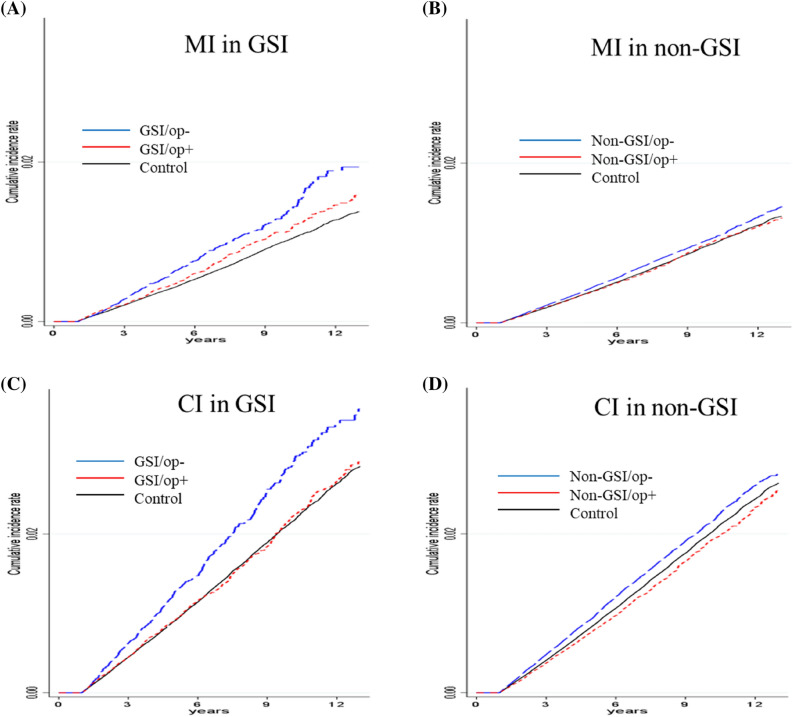
Cumulative incidences of myocardial infarction (MI) and cerebral infarction (CI) according to gallstone-related infection (GSI) and cholecystectomy. (**A**,**B**) myocardial infarction, (**A**) gallstone-related infection (GSI), (**B**) non-GSI. (**C**,**D**) cerebral infarction, (**C**) GSI, (**D**) non-GSI. op, cholecystectomy.

The cumulative incidence of MI or CI according to cholecystectomy in both the GSI and non-GSI groups was presented (Fig. 2). In both the GSI and non-GSI groups, MI or CI incidences were reduced in patients who had undergone cholecystectomy compared to those who did not. However, the GSI group showed a higher risk reduction after cholecystectomy than the non-GSI group.

The IRR (95% CI) and aHR (95% CI) of MI or CI were analyzed according to cholecystectomy in the GSI and non-GSI groups (Table 2). In the GSI group, the IRRs and aHR of MI were lower in patients who underwent cholecystectomy (1.15 [1.03–1.28] and 1.17 [1.05–1.29], respectively) than in those who did not (1.40 [1.23–1.60] and 1.32 [1.15–1.50], respectively). However, in the non-GSI group, the IRR and aHR of MI were nearly matched between patients who underwent cholecystectomy (1.01 [0.94–1.08] and 1.07 [1.00–1.15], respectively) and those who did not (1.10 [1.04–1.15] and 1.07 [1.02–1.12], respectively). Risk reduction rates in the GSI and non-GSI groups were 11% and 0%, respectively.

The IRRs and aHR of CI were lower in patients who underwent cholecystectomy (1.01 [0.93–1.09] and 1.04 [0.96–1.12], respectively) than in those who did not (1.32 [1.20–1.44] and 1.24 [1.13–1.36], respectively) in the GSI group. Although the IRR and aHR of CI were lower in patients who underwent cholecystectomy (0.95 [0.90–1.00] and 1.01 [0.96–1.07], respectively) than in those who did not (1.09 [1.05–1.13] and 1.06 [1.03–1.10], respectively), its effect was minimal in the non-GSI group. Risk reduction rates in the GSI and non-GSI groups were 16% and 5%, respectively.

The aHR of each confounding factor are presented in Table 3 and Supplement 1. Hypertension, pulse pressure, fasting plasma glucose (FPG), body mass index (BMI), cholesterol, and smoking were dose-responsive risk factors for MI or CI. Cholesterol and smoking were associated with a higher risk of MI than CI, whereas hypertension was associated with a higher risk of CI than MI. Pulse pressure, FPG and BMI revealed similar risks for MI and CI. Physical activity reduced the risk of MI and CI, while alcohol consumption being negatively associated with MI but not CI.

**Table 3:** Multivariate Cox regression analysis of risk factors associated with development of myocardial infarction and cerebral infarction.

Parameters	Myocardial infarction			Cerebral infarction		
	Gallstones	GSI	Non-GSI	Gallstones	GSI	Non-GSI
Cholecystectomy (−)	1.10 (1.05, 1.15)*	1.32 (1.15, 1.50)	1.07 (1.02, 1.12)	1.08 (1.05, 1.12)	124 (1.13, 1.36)	106 (1.03, 1.10)
Cholecystectomy (+)	1.10 (1.03, 1.16)	1.17 (1.05, 1.29)	1.07 (1.00, 1.15)	1.02 (0.98, 1.06)	1.04 (0.96, 1.12)	1.01 (0.96, 1.07)
**SBP/DBP**						
< 120/ < 80	1.00	1.00	1.00	1.00	1.00	1.00
120–129/ < 80	1.05 (1.00, 1.09)	1.04 (0.94, 1.15)	1.05 (1.00, 1.10)	1.06 (1.00, 1.09)	1.06 (1.99, 1.15)	1.05 (1.02, 1.09)
130–139/80–89	1.16 (1.12, 1.19)	1.09 (1.01, 1.17)	1.17 (1.13, 1.21)	1.21 (1.18, 1.24)	1.25 (1.19, 1.32)	1.20 (1.17, 1.23)
140–179/90–119	1.29 (1.24, 1.34)	1.21 (1.11, 1.32)	1.30 (1.25, 1.36)	1.41 (1.38, 1.45)	1.43 (1.34, 1.52)	1.41 (1.37, 1.45)
180+/120+	1.68 (1.52, 1.86)	1.53 (1.20, 1.96)	1.71 (1.54, 1.91)	1.94 (1.82, 2.07)	1.73 (1.48, 2.03)	1.98 (1.84, 2.12)
**Pulse pressure**						
< 40	1.00	1.00	1.00	1.00	1.00	1.00
40–59	1.07 (1.03, 1.12)	1.05 (0.95, 1.15)	1.08 (1.03, 1.13)	1.09 (1.06, 1.13)	1.09 (1.01, 1.17)	1.09 (1.06, 1.13)
≥ 60	1.13 (1.07, 1.18)	1.03 (0.92, 1.16)	1.15 (1.09, 1.21)	1.14 (1.10, 1.18)	1.14 (1.05, 1.23)	1.13 (1.09, 1.18)
**FPG (mg/dl)**						
< 100	1.00	1.00	1.00	1.00	1.00	1.00
100–125	1.07 (1.05, 1.10)	1.10 (1.04, 1.17)	1.07 (1.04, 1.10)	1.06 (1.04, 1.08)	1.05 (1.01, 1.10)	1.06 (1.04, 1.09)
≥ 126	1.65 (1.60, 1.71)	1.64 (1.52, 1.77)	1.66 (1.60, 1.72)	1.60 (1.56, 1.63)	1.58 (1.50, 1.67)	1.60 (1.57, 1.64)
**BMI**						
< 18.5	1.00	1.00	1.00	1.00	1.00	1.00
18.5–25.0	1.04 (0.96, 1.13)	1.02 (0.85, 1.22)	1.04 (0.96, 1.14)	1.09 (1.04, 1.15)	1.10 (0.97, 1.23)	1.09 (1.03, 1.15)
≥ 25.0	1.08 (1.06, 1.11)	1.07 (1.02, 1.13)	1.09 (1.06, 1.12)	1.02 (1.01, 1.04)	1.00 (0.97, 1.04)	1.03 (1.01, 1.04)
**Cholesterol (mg/dl)**						
< 18.5	1.00	1.00	1.00	1.00	1.00	1.00
18.5–25.0	1.21 (1.18, 1.24)	1.22 (1.15, 1.29)	1.21 (1.18, 1.24)	1.05 (1.03, 1.07)	1.11 (1.06, 1.15)	1.04 (1.02, 1.06)
≥ 25.0	1.53 (1.49, 1.58)	1.55 (1.44, 1.68)	1.53 (1.47, 1.58)	1.11 (1.09, 1.14)	1.14 (1.08, 1.21)	1.11 (1.08, 1.13)
**Smoking (pack/year)**						
None	1.00	1.00	1.00	1.00	1.00	1.00
1–9	1.22 (1.16, 1.28)	1.24 (1.10, 1.40)	1.21 (1.15, 1.28)	1.14 (1.09, 1.18)	1.18 (1.08, 1.30)	1.13 (1.08, 1.18)
10–19	1.45 (1.40, 1.51)	1.44 (10.10, 1.58)	1.46 (1.40, 1.52)	1.26 (1.23, 1.30)	1.24 (1.16, 1.33)	1.27 (1.23, 1.31)
20–29	1.62 (1.56, 1.69)	1.63 (1.48, 1.80)	1.62 (1.55, 1.69)	1.38 (1.34, 1.43)	1.39 (1.29, 1.49)	1.38 (1.34, 1.43)
30–39	1.67 (1.59, 1.77)	1.57 (1.38, 1.78)	1.70 (1.60, 1.80)	1.37 (1.32, 1.43)	1.40 (1.27, 1.54)	1.37 (1.30, 1.43)
40 +	1.81 (1.73, 1.90)	1.82 (1.63, 2.03)	1.81 (1.72, 1.91)	1.50 (1.45, 1.56)	1.46 (1.35, 1.59)	1.51 (1.46, 1.57)
**Alcohol drinking**						
None	1.00	1.00	1.00	1.00	1.00	1.00
< one/month	0.75 (0.73, 0.78)	0.78 (0.72, 0.84)	0.74 (0.72, 0.77)	0.97 (0.95, 1.00)	1.00 (0.94, 1.06)	0.97 (0.94, 1.00)
< one/week	0.74 (0.70, 0.78)	0.70 (0.60, 0.81)	0.74 (0.70, 0.79)	0.97 (0.93, 1.01)	0.94 (0.85, 1.04)	0.98 (0.93, 1.02)
≥ one/week	0.60 (0.58, 0.63)	0.64 (0.58, 0.71)	0.60 (0.57, 0.63)	1.05 (1.02, 1.08)	1.03 (0.96, 1.10)	1.05 (1.02, 1.09)
**Physical activity**						
None	1.00	1.00	1.00	1.00	1.00	1.00
1–2/week	0.90 (0.87, 0.92)	0.87 (0.81, 0.93)	0.90 (0.87, 0.93)	0.82 (0.80, 0.84)	0.80 (0.76, 0.84)	0.82 (0.81, 0.84)
3–4/week	0.83 (0.80, 0.86)	0.82 (0.75, 0.90)	0.83 (0.80, 0.87)	0.76 (0.74, 0.78)	0.78 (0.73, 0.83)	0.76 (0.73, 0.78)
5–6/week	0.84 (0.79, 0.89)	0.82 (0.72, 0.94)	0.84 (0.79, 0.89)	0.77 (0.74, 0.81)	0.76 (0.69, 0.84)	0.78 (0.74, 0.81)
Every day	0.91 (0.87, 0.95)	0.90 (0.81, 1.00)	0.91 (0.87, 0.96)	0.87 (0.85, 0.90)	0.83 (0.78, 0.90)	0.88 (0.85, 0.91)

*, hazard ratio (95% confidence interval); †, number in parenthesis means the amount of alcohol consumption in women; GSI, gallstone-related infection; SBP/DBP, systolic blood pressure/diastolic blood pressure; BMI, body mass index; FPG, fasting plasma glucose.

### Subgroup analysis of MI or CI risks by both sexes and by different ages

**Figure 3:**
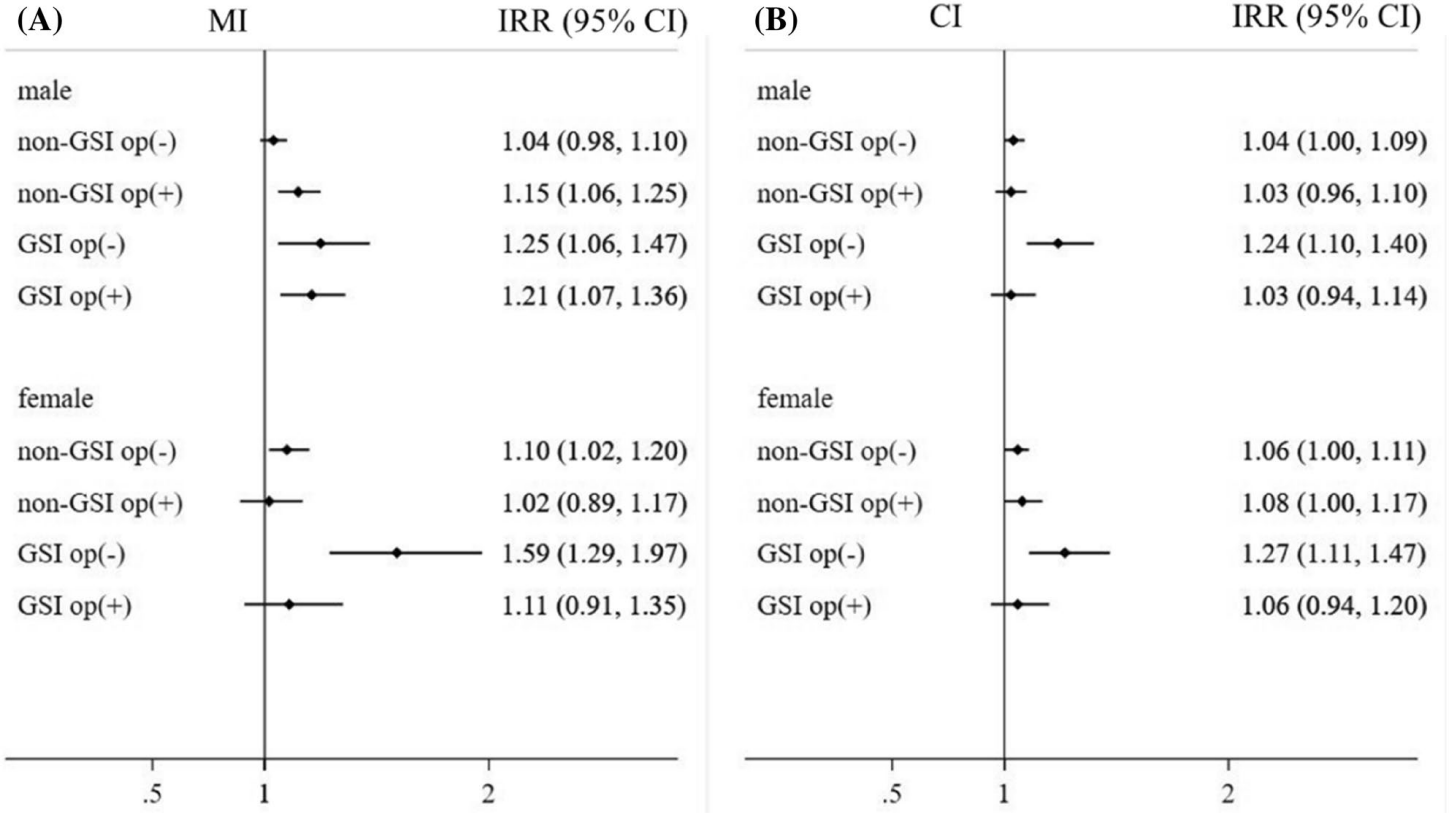
Incidence rate ratios of myocardial infarction (MI) and cerebral infarction (CI) according to gallstone-related infection (GSI) and cholecystectomy in males and females. (**A**) MI, (**B**)  CI. op, cholecystectomy.

**Figure 4:**
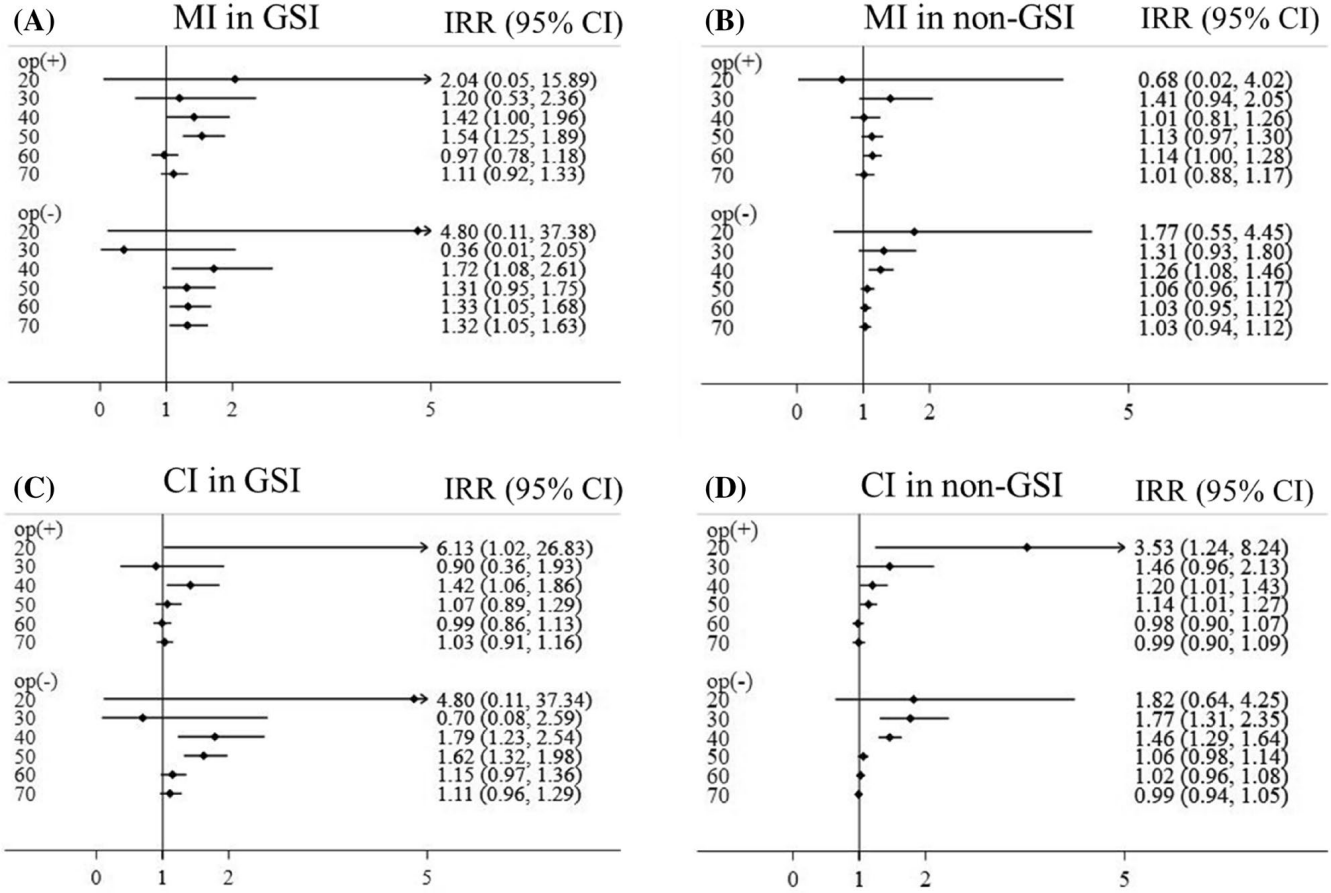
Incidence rate ratios of myocardial infarction (MI) and cerebral infarction (CI) according to gallstone-related infection (GSI) and cholecystectomy in age groups. (**A**,**B**) myocardial infarction, (**A**) GSI, (**B**) non-GSI. (**C**,**D**) cerebral infarction, (**C**) GSI, (**D**) non-GSI. op, cholecystectomy.

A subgroup analysis was performed for both males and females. Females had a higher risk and higher risk reduction rate after cholecystectomy for MI than males, while there were no differences in CI risks between sexes (Fig. 3). Subgroup analysis was performed for different age groups. Risks of MI or CI and risk reduction rates by cholecystectomy were higher in younger patients than in older patients (Fig. 4).

## Discussion

The positive association between gallstones and CVD has recently received much attention because of the increasing role of inflammation in the development of CVD and gallstones. In addition to the subclinical inflammatory process, acute bacterial infection associated with gallstones may accelerate atherosclerosis in the vasculature. We carried out a risk analysis to investigate the CVD risk in the GSI or non-GSI groups treated by cholecystectomy. In this study, we demonstrated that CVD risks and risk changes due to treatment are determined by the presence or absence of GSI. The GSI group had greater risks of MI or CI and higher risk reduction by cholecystectomy than the non-GSI group. To the best of our knowledge, this is the first study to demonstrate that CVD risk is strongly associated with GSI and is partially reduced by cholecystectomy in patients with GSI.

The role of inflammation in CVD risk has been emphasized in recent years. Control of the inflammatory reaction is targeted as a strategy for atheroprotection^24^. We hypothesized that CVD risk among gallstone patients increases under GSI conditions. Gram-negative bacteremia frequently occurs in patients with GSI, which produces LPS or circulating endotoxins. These inflammatory mediators accelerate atherosclerosis or vasculopathy via cytokines from human vascular endothelial and smooth muscle cells^25^. In this study, the risk of MI or CI increased by 32% and 24%, respectively, in the GSI group compared to controls, while they were nearly equal to controls in the non-GSI group. Studies regarding CVD risk in GSI and non-GSI groups have reported mixed results^5,17,18^. In one study, risk of ischemic stroke increased by 7% in symptomatic gallstones compared with asymptomatic gallstones, while hemorrhagic stroke showed similar results^17^. On the other hand, CVD risk was similar between severe and non-severe gallstone patients in another study^18^. They defined symptomatic gallstones as acute cholecystitis, acute cholangitis, biliary pancreatitis, or those treated by surgery or endoscopic intervention^18^. Moreover, recent meta-analysis^5^ reported that CVD risk was higher in screen-detected gallstones than in symptomatic gallstones with 35% and 21% increased risk, respectively, with reference to controls. These unmatched results may be caused by incomplete adjustment of other risk factors such as obesity, physical inactivity, high blood pressure, high blood glucose level, alcohol drinking or smoking.

In the present study, cholecystectomy had a greater effect on the CVD risk in the GSI group than in the non-GSI group. Cholecystectomy partially reduced the increased risk of CVD in the GSI group. Attenuation of systemic inflammation by cholecystectomy may have a role in reducing CVD risk^17^. Risk reduction by cholecystectomy was greater for CI than for MI in the GSI group, with 16.1% and 11.4%, respectively. However, the original risk of CVD and the risk reduction by cholecystectomy were negligible in the non-GSI group. Studies regarding cholecystectomy and CVD risk factors are rare and have reported mixed results. A population-based study in Taiwan revealed that stroke risk decreased in gallstone patients who underwent cholecystectomy compared with those who did not. Risk reduction rates were 46% and 25% for symptomatic gallstones and asymptomatic gallstones, respectively^17^. However, several studies have reported that cholecystectomy for gallstones did not influence CVD risks^19–21^. Moreover, CVD risk was higher in case of gallstones treated by cholecystectomy compared to screening-detected gallstones^19^ or gallstones treated conservatively^20^. However, they did not compare CVD risks separately in GSI and non-GSI groups nor adjustment by severity of inflammation. Cholecystectomy is usually indicated for biliary pain and gallstone-related complications. Therefore, patients who require cholecystectomy may have more complicated gallstones. Therefore, the increased CVD risk in the cholecystectomy group was not due to cholecystectomy itself but due to confounding variables related to high CVD risk.

In the present study, CVD risk among gallstone patients was higher in younger than in older patients. This finding was consistent with a recent meta-analysis^7^. Because younger people had a low prevalence of gallstones and fewer other CVD risks, the effect of gallstones or cholecystectomy seemed to be stronger in the younger group^18^. Theses findings suggested that younger patients with gallstones should be given more attention for the prevention of CVD. In addition, the risk of MI was higher in females than in males, whereas the risk of CI was similar between the sexes. These findings were consistent with those of previous studies, where CVD risks were higher in females than in males among gallstone patients^5,7,18,26,27^. The explanation for these results is not clear, but we speculate that gallstones or cholecystectomy seemed to have a stronger risk of MI in females because of fewer other risk factors and a lower incidence of MI compared to males. No previous studies have performed subgroup analysis for the effect of cholecystectomy in terms of sex or age. This is the first study to demonstrate that risk reduction by cholecystectomy was higher in younger patients and females compared to older patients and males.

Our data were compatible with previous studies showing that obesity^28,29^, hyperlipidemia^21^, diabetes mellitus, hypertension^30^, smoking^31,32^ and long sedentary periods^33^ are associated with gallstones and CVD^34^. These cardio-metabolic factors were revealed as dose-responsive predictors of both MI and CI, with a slight difference between them. As in a previous study^35^, hypertension was associated with a higher risk of CI than MI, whereas cholesterol and smoking showed the opposite effect. In this study, the adjusted risk (aHR) of gallstones for CVD development was lower than crude risk (IRR). CVD risk among gallstone patients was usually lower with complete adjustment of confounding factors, longer follow-up, and a larger study compared to their counterparts^26^. Therefore, adjustment of other risk factors is needed to evaluate the real effects of gallstones on CVD development.

Disease patterns of CVD are slightly different in Eastern and Western populations. Coronary heart disease is prevalent in Western people, while stroke is prevalent in Eastern people^35,36^. This study of Korean people revealed that CI incidence was 2-fold higher than MI and the risk reduction rate by cholecystectomy was higher for CI than MI. These differences between MI and CI could be explained by regional or racial variations with regards to the risk factors of MI or CI. Therefore, our results are limited to Asian populations and further studies are needed for other ethnicities.

We selected controls by matching age, sex, and visit frequency as outpatients. Matched clinic visit frequency is important to reduce selection bias in terms of economic status, interest in health, and detection bias. Using this method, we selected a control group to investigate the real effects of gallstones or cholecystectomy on the risk of MI or CI.

We identified all patients hospitalized with a diagnosis of first-time MI (I21) or CI (I63) using the International Classification of Disease (ICD)-10 codes. The incidences of MI or CI in this study were consistent to those in previous studies of South Korea^37,38^. We also confirmed that patients and controls had similar all-cause mortality rates (Supplement 3). We suggest that patients of MI or CI included in this study are representative groups.

In this study, we defined GSI group as patients diagnosed with acute cholecystitis or acute cholangitis associated with gallstones using ICD-10 codes. Because the proportion of acute biliary pancreatitis was less than 1% and was not related to bacterial infection, we excluded acute biliary pancreatitis in the GSI group. The ICD-10 code for gallstones has been validated in a previous study^39^.

This study has several limitations. First, we could not identify the clinical features of one third of patients, who did not receive cholecystectomy in spite of GSI. Patients with acute cholecystitis are usually recommended to be treated by cholecystectomy. However, in clinical practice, some patients did not receive cholecystectomy^40^. Similar to our result, 22% of symptomatic patients did not receive cholecystectomy in Taiwan’s study^27^. These patients have possibilities that they are recovered from mild disease or too sick to receive cholecystectomy. The proportion of GSI among gallstones was 17% in this study, which was higher than previous study^41^. Mild GSI may be more included in this study. In addition, some patients are too sick to receive operation. To overcome such potential bias, we excluded patients who died within 1 year of diagnosis. Patients survived more than 1 year after GSI are usually tolerable to cholecystectomy^42^. We suggest that patients with severe comorbidity not to receive cholecystectomy are not great in this study. Second, we selected control group among people who performed health surveillance, who do not have gallstone related ICD-10 codes or cholecystectomy. However, there is still possibility, that included asymptomatic gallstones. In South Korea, prevalence of gallstones in a general health screened population has been reported as 2–4%^43,44^. We supposed that asymptomatic gallstone in control group could not influence on the results. Third, there were unadjusted risk factors for CVD and gallstones, such as insulin resistance, lipid-lowering drugs, inflammatory mediators, nonalcoholic fatty liver disease^45,46^, and the gut microbiota. Despite these limitations, this is the first study to extensively evaluate the effects of cholecystectomy on the risk of MI or CI by adjusting for confounding factors in a large population-based cohort.

In conclusion, cholecystectomy for GSI reduces the risk of MI or CI independent of other risk factors, and it was greater in females and younger patients. However, it had minimal effect on CVD in the non-GSI group. Among patients with GSI, younger people and females who have risk factors for CVD need close monitoring for CVD development. Cholecystectomy is selectively recommended in patients with prior or current biliary tract infection to reduce the risk of ischemic CVD and recurrence of biliary complications.

## Methods

### Data sources

The KNHI and KNHSP databases were used in this study. The KNHI is a mandatory health insurance program that covers 97.1% of the Korean population^47^ comprising approximately 50 million individuals. Comprehensive information regarding the medical services provided to patients, such as diagnosis, demographics, prescriptions, surgeries, tests, and imaging studies were recorded in the KNHI database. Patient diagnoses were documented in accordance with the ICD-10. KNHI insurance subscribers are recommended to undergo standardized general health screenings biennially under the KNHSP, data of which are recorded in the KNHSP database. The KNHSP data contain information on patients’ lifestyle and behavioral factors such as physical activity, alcohol consumption, and smoking status obtained during health checkups and questionnaires. It also includes laboratory test results and measurements, including BMI, SBP and DBP, FPG, and cholesterol levels. We used the latest health check-up data, which obtained within 2 years before enrollment.

### Study population

We extracted all patients with gallstones (n = 1,269,752) from the KNHI data who were registered between January 1, 2005, and December 31, 2017 according to the ICD-10 code (K80.0–80.2). We excluded patients with combined bile duct stones (K80.3–80.5, n = 275,528), who underwent cholecystectomy (Q7370) prior to or after 1 year of diagnosis (n = 56,089), MI or CI prior to or within 1 year of diagnosis (n = 10,200 or n = 27,477, respectively), who died within 1 year of diagnosis (n = 23,139), who were diagnosed with cancer before or within 1 year of diagnosis (n = 31,300), and who had incomplete health surveillance data (n = 354,752). Finally, 491,267 participants were included in this study. Patients were classified into GSI and non-GSI groups. The GSI group included patients with acute cholecystitis (K80.0, K80.1, or K80.0–80.2 and K81.x, n = 80,076) or acute cholangitis (K80.0–80.2 and K83.0, n = 4391) among gallstone patients. The GSI and non-GSI groups were further subdivided into individuals treated with or without cholecystectomy. Controls were defined as general people, who did not have gallstone related ICD-10 codes (K80–83) or cholecystectomy. They were selected among health surveillance people with 1:10 matched age, sex, and visit frequency as outpatients within one year for each of the GSI and non-GSI groups (n = 844,670 and n = 4,068,000, respectively).

### Assessment of MI and CI

We identified MI and CI patients, who had history of hospital admission for their respective ICD-10 codes (I21 and I63, respectively). Patients were followed up until December 31, 2016, and December 31, 2017, to detect MI or CI and identify their vital status. Information regarding the vital status of each individual was obtained from Statistics Korea. For a given case, the person-year at risk was calculated as the period between the date of enrollment and the date of MI or CI diagnosis or the exit date.

### Outcomes and statistical analysis

The characteristics of the GSI and non-GSI groups who did or did not undergo cholecystectomy were compared with their corresponding controls using the standard difference of means. The cumulative incidence curves of MI and CI were prepared according to GSI and non-GSI groups. The incidence of MI or CI with 95% CI was measured as the number of cases per 10^4^ person-years. We measured the IRR of MI or CI in each group, using controls as a reference. Cox proportional hazard analysis was performed to examine the relationship between MI or CI incidence and explanatory factors such as age (20–29, 30–39, 40–49, 50–59, 60–69, 70+ years old), sex, systolic and diastolic blood pressure (SBP and DBP) (< 120/ < 80, 120–129/ < 80, 130–139/80–89, 140–179/90–119, 180 + /120 + mmHg), pulse pressure (< 40, 40–59, 60 + mmHg), FPG (< 100, 100–125, 126 + mg/dl), cholesterol (< 200, 200–239, 240 + mg/dl), BMI (< 18.5, 18.5–25.0, 25.0 + kg/m^2^), smoking (none, 1–9, 10–19, 20–29, 30–39, 40 + pack/year), alcohol drinking (none, < one/month, < one/week and ≥ one/week) and physical activity (none, 1–2/week, 3–4/week, 5–6/week, every day). We adjusted the visit frequency as an outpatient within one year in patients and matched controls to eliminate detection bias. The results are expressed in terms of the aHR of the incidence with 95% CIs. A subgroup analysis was performed for both sexes and age groups. Statistical analyses were performed using the Stata/MP2 software (version 13.1; StataCorp, College Station, TX, USA). Statistical significance was defined as a *P* value < 0.05.

### Ethical considerations

The study was reviewed and approved by the Ethics Committee of Korea University (KUIRB-2020-0021-01). All methods were performed in accordance with relevant regulations. Informed consent was waived because the KNHSP or KNHI database contains publicly available anonymized data.

## Supplementary Information

Supplementary Information.

## Data Availability

The datasets generated and analyzed during this study are available from the corresponding author on reasonable request.
