# Supplementary material for: Cholecystectomy reduces the risk of myocardial and cerebral infarction in patients with gallstone-related infection
Source: Sci Rep. 2022 Oct 6;12:16749. doi: 10.1038/s41598-022-20700-y (PMC9537563; doi:10.1038/s41598-022-20700-y)
Supplement: Supplementary file 1 — Supplementary Information. [file 41598_2022_20700_MOESM1_ESM.docx]

Supplement 1. Univariate Cox regression analysis of risk factors associated with development of myocardial infarction and cerebral infarction

| Parameters | Myocardial infarction | | | Cerebral infarction | | |
| --- | --- | --- | --- | --- | --- | --- |
|  | Gallstones | GSI | Non-GSI | Gallstones | GSI | Non-GSI |
| Cholecystectomy (-) | 1.10 (1.05, 1.15)* | 1.32 (1.16, 1.51) | 1.07 (1.02, 1.13) | 1.09 (1.05, 1.12) | 1.24 (1.13, 1.37) | 1.07 (1.03, 1.11) |
| Cholecystectomy (+) | 1.13 (1.06, 1.20) | 1.20 (1.08, 1.34) | 1.10 (1.03, 1.18) | 1.03 (0.99, 1.08) | 1.05 (0.97, 1.14) | 1.03 (0.98, 1.08) |
| SBP / DBP |  |  |  |  |  |  |
| <120 / <80 | 1.00 | 1.00 | 1.00 | 1.00 | 1.00 | 1.00 |
| 120-129 / <80 | 1.08 (1.04, 1.13) | 1.06 (0.96, 1.17) | 1.09 (1.04, 1.14) | 1.09 (1.06, 1.12) | 1.10 (1.02, 1.18) | 1.09 (1.05, 1.12) |
| 130-139 / 80-89 | 1.20 (1.16, 1.23) | 1.11 (1.03, 1.19) | 1.21 (1.17, 1.25) | 1.25 (1.22, 1.28) | 1.29 (1.22, 1.36) | 1.24 (1.21, 1.27) |
| 140-179 / 90-119 | 1.38 (1.34, 1.43) | 1.26 (1.16, 1.36) | 1.41 (1.36, 1.46) | 1.52 (1.48, 1.55) | 1.53 (1.45, 1.61) | 1.52 (1.48, 1.55) |
| 180+ /120+ | 1.89 (1.72, 2.09) | 1.65 (1.30, 2.09) | 1.96 (1.76, 2.17) | 2.19 (2.05, 2.32) | 1.96 (1.68, 2.29) | 2.23 (2.08, 2.38) |
| Pulse Pressure |  |  |  |  |  |  |
| <40 | 1.00 | 1.00 | 1.00 | 1.00 | 1.00 | 1.00 |
| 40-59 | 1.12 (1.08, 1.17) | 1.08 (0.99, 1.19) | 1.13 (1.08, 1.18) | 1.15 (1.12, 1.19) | 1.15 (1.08, 1.23) | 1.15 (1.12, 1.19) |
| ≥60 | 1.35 (1.30, 1.41) | 1.20 (1.08, 1.33) | 1.39 (1.32, 1.45) | 1.44 (1.39, 1.48) | 1.42 (1.32, 1.54) | 1.44 (1.39, 1.49) |
| FPG (mg/dl) |  |  |  |  |  |  |
| <100 | 1.00 | 1.00 | 1.00 | 1.00 | 1.00 | 1.00 |
| 100-125 | 1.11 (1.08, 1.13) | 1.13 (1.06, 1.20) | 1.10 (1.07, 1.13) | 1.09 (1.07, 1.11) | 1.08 (1.04, 1.13) | 1.10 (1.08, 1.12) |
| ≥126 | 1.74 (1.68, 1.79) | 1.71 (1.58, 1.84) | 1.74 (1.69, 1.81) | 1.68 (1.65, 1.72) | 1.66 (1.58, 1.75) | 1.69 (1.65, 1.73) |
| BMI |  |  |  |  |  |  |
| <18.5 | 1.00 (0.93, 1.08) | 1.00 (0.84, 1.20) | 1.00 (0.92, 1.09) | 1.08 (1.06, 1.11) | 1.06 (0.95, 1.19) | 1.06 (1.00, 1.12) |
| 18.5-25.0 | 1.00 | 1.00 | 1.00 | 1.00 | 1.00 | 1.00 |
| ≥25.0 | 1.15 (1.13, 1.18) | 1.13 (1.07, 1.19) | 1.16 (1.13, 1.19) | 1.09 (1.076, 1.10) | 1.07 (1.03, 1.11) | 1.09 (1.07, 1.11) |
| Cholesterol (mg/dl) |  |  |  |  |  |  |
| <18.5 | 1.00 | 1.00 | 1.00 | 1.00 | 1.00 | 1.00 |
| 18.5-25.0 | 1.23 (1.20, 1.26) | 1.23 (1.16, 1.30) | 1.22 (1.19, 1.26) | 1.06 (1.04, 1.08) | 1.12 (1.07, 1.16) | 1.05 (1.03, 1.07) |
| ≥25.0 | 1.61 (1.56, 1.66) | 1.63 (1.51, 1.76) | 1.61 (1.55, 1.66) | 1.17 (1.14, 1.19) | 1.20 (1.13, 1.26) | 1.16 (1.13, 1.19) |
| Smoking (pack/year) |  |  |  |  |  |  |
| none | 1.00 | 1.00 | 1.00 | 1.00 | 1.00 | 1.00 |
| 1~9 | 1.13 (1.07, 1.19) | 1.16 (1.02, 1.30) | 1.12 (1.06, 1.19) | 1.11 (1.06, 1.15) | 1.15 (1.06, 1.26) | 1.10 (1.05, 1.15) |
| 10~19 | 1.36 (1.31, 1.41) | 1.35 (1.23, 1.48) | 1.36 (1.30, 1.42) | 1.25 (1.21, 1.29) | 1.22 (1.14, 1.31) | 1.26 (1.22, 1.30) |
| 20~29 | 1.52 (1.46, 1.58) | 1.54 (1.40, 1.69) | 1.51 (1.45, 1.58) | 1.40 (1.35, 1.44) | 1.39 (1.30, 1.50) | 1.40 (1.35, 1.44) |
| 30~39 | 1.58 (1.50, 1.67) | 1.49 (1.31, 1.70) | 1.60 (1.51, 1.70) | 1.40 (1.34, 1.46) | 1.42 (1.29, 1.56) | 1.39 (1.33, 1.46) |
| 40+ | 1.74 (1.66, 1.82) | 1.76 (1.58, 1.97) | 1.73 (1.64, 1.82) | 1.55 (1.50, 1.61) | 1.50 (1.39, 1.63) | 1.57 (1.51, 1.63) |
| Alcohol drinking |  |  |  |  |  |  |
| none | 1.00 | 1.00 | 1.00 | 1.00 | 1.00 | 1.00 |
| < one/month | 0.78 (0.75, 0.80) | 0.80 (0.74, 0.87) | 0.77 (0.74, 0.80) | 0.99 (0.96, 1.01) | 1.01 (0.95, 1.07) | 0.98 (0.96, 1.01) |
| < one/week | 0.81 (0.77, 0.86) | 0.76 (0.66, 0.88) | 0.82 (0.77, 0.87) | 1.02 (0.98, 1.06) | 0.98 (0.89, 1.09) | 1.02 (0.98, 1.07) |
| ≥ one/week | 0.71 (0.68, 0.74) | 0.75 (0.68, 0.82) | 0.71 (0.68, 0.74) | 1.19 (1.15, 1.22) | 1.16 (1.08, 1.24) | 1.19 (1.15, 1.23) |
| Physical activity |  |  |  |  |  |  |
| none | 1.00 | 1.00 | 1.00 | 1.00 | 1.00 | 1.00 |
| 1~2/week | 0.87 (0.85, 0.90) | 0.85 (0.79, 0.91) | 0.88 (0.85, 0.90) | 0.81 (0.79, 0.82) | 0.79 (0.75, 0.83) | 0.81 (0.79, 0.83) |
| 3~4/week | 0.80 (0.77, 0.83) | 0.79 (0.72, 0.87) | 0.80 (0.77, 0.84) | 0.75 (0.73, 0.77) | 0.76 (0.72, 0.82) | 0.74 (0.72, 0.77) |
| 5~6/week | 0.81 (0.77, 0.86) | 0.80 (0.70, 0.92) | 0.81 (0.76, 0.86) | 0.77 (0.73, 0.80) | 0.75 (0.68, 0.83) | 0.77 (0.73, 0.80) |
| every day | 0.90 (0.86, 0.94) | 0.89 (0.80, 0.99) | 0.90 (0.86, 0.95) | 0.88 (0.85, 0.91) | 0.84 (0.78, 0.90) | 0.89 (0.86, 0.92) |

*, hazard ratio (95% confidence interval); †, number in parenthesis means the amount of alcohol consumption in women; GSI, gallstone-related infection; SBP/DBP, systolic blood pressure/diastolic blood pressure; BMI, body mass index; FPG, fasting plasma glucose.

Supplement 2. Multivariate Cox regression analysis of risk factors associated with development of myocardial infarction and cerebral infarction (dichotomous variables)

| Parameters | Myocardial infarction | | | Cerebral infarction | | |
| --- | --- | --- | --- | --- | --- | --- |
|  | Gallstones | GSI | Non-GSI | Gallstones | GSI | Non-GSI |
| Cholecystectomy (-) | 1.10 (1.05, 1.15)* | 1.32 (1.15, 1.50) | 1.07 (1.02, 1.12) | 1.08 (1.05, 1.12) | 124 (1.13, 1.36) | 106 (1.03, 1.10) |
| Cholecystectomy (+) | 1.10 (1.03, 1.16) | 1.17 (1.05, 1.29) | 1.07 (1.00, 1.15) | 1.02 (0.98, 1.06) | 1.04 (0.96, 1.12) | 1.01 (0.96, 1.07) |
| Hypertension |  |  |  |  |  |  |
| No | 1.00 | 1.00 | 1.00 | 1.00 | 1.00 | 1.00 |
| Yes | 1.19 (1.15, 1.22) | 1.16 (1.09, 1.24) | 1.19 (1.16, 1.23) | 1.26 (1.24, 1.29) | 1.24 (1.18, 1.30) | 1.27 (1.24, 1.29) |
| Diabetes |  |  |  |  |  |  |
| No | 1.00 | 1.00 | 1.00 | 1.00 | 1.00 | 1.00 |
| Yes | 1.62 (1.57, 1.67) | 1.59 (1.48, 1.71) | 1.63 (1.57, 1.68) | 1.57 (1.54, 1.60) | 1.56 (1.49, 1.64) | 1.57 (1.54, 1.61) |
| Obesity |  |  |  |  |  |  |
| No | 1.00 | 1.00 | 1.00 | 1.00 | 1.00 | 1.00 |
| Yes | 1.12 (1.09, 1.15) | 1.11 (1.05, 1.17) | 1.12 (1.10, 1.15) | 1.05 (1.03, 1.07) | 1.03 (0.99, 1.07) | 1.05 (1.03, 1.07) |
| Hyperlipidemia |  |  |  |  |  |  |
| No | 1.00 | 1.00 | 1.00 | 1.00 | 1.00 | 1.00 |
| Yes | 1.31 (1.28, 1.34) | 1.32 (1.25, 1.39) | 1.31 (1.28, 1.34) | 1.07 (1.06, 1.09) | 1.12 (1.08, 1.17) | 1.06 (1.05, 1.08) |
| Smoking (pack/year) |  |  |  |  |  |  |
| None | 1.00 | 1.00 | 1.00 | 1.00 | 1.00 | 1.00 |
| Mild | 1.06 (1.02, 1.10) | 1.05 (0.97, 1.15) | 1.06 (1.02, 1.11) | 0.96 (0.93, 0.98) | 0.98 (0.92, 1.04) | 0.95 (0.92, 0.98) |
| Heavy | 1.80 (1.74, 1.85) | 1.81 (1.69, 1.95) | 1.79 (1.73, 1.85) | 1.54 (1.50, 1.57) | 1.51 (1.43, 1.59) | 1.54 (1.51, 1.58) |
| Alcohol drinking |  |  |  |  |  |  |
| none | 1.00 | 1.00 | 1.00 | 1.00 | 1.00 | 1.00 |
| < one/month | 0.75 (0.73, 0.78) | 0.78 (0.72, 0.84) | 0.74 (0.72, 0.77) | 0.97 (0.95, 1.00) | 1.00 (0.94, 1.06) | 0.97 (0.94, 1.00) |
| < one/week | 0.74 (0.70, 0.78) | 0.70 (0.60, 0.81) | 0.74 (0.70, 0.79) | 0.97 (0.93, 1.01) | 0.94 (0.85, 1.04) | 0.98 (0.93, 1.02) |
| ≥ one/week | 0.60 (0.58, 0.63) | 0.64 (0.58, 0.71) | 0.60 (0.57, 0.63) | 1.05 (1.02, 1.08) | 1.03 (0.96, 1.10) | 1.05 (1.02, 1.09) |
| Physical activity |  |  |  |  |  |  |
| none | 1.00 | 1.00 | 1.00 | 1.00 | 1.00 | 1.00 |
| 1~2/week | 0.90 (0.87, 0.92) | 0.87 (0.81, 0.93) | 0.90 (0.87, 0.93) | 0.82 (0.80, 0.84) | 0.80 (0.76, 0.84) | 0.82 (0.81, 0.84) |
| 3~4/week | 0.83 (0.80, 0.86) | 0.82 (0.75, 0.90) | 0.83 (0.80, 0.87) | 0.76 (0.74, 0.78) | 0.78 (0.73, 0.83) | 0.76 (0.73, 0.78) |
| 5~6/week | 0.84 (0.79, 0.89) | 0.82 (0.72, 0.94) | 0.84 (0.79, 0.89) | 0.77 (0.74, 0.81) | 0.76 (0.69, 0.84) | 0.78 (0.74, 0.81) |
| every day | 0.91 (0.87, 0.95) | 0.90 (0.81, 1.00) | 0.91 (0.87, 0.96) | 0.87 (0.85, 0.90) | 0.83 (0.78, 0.90) | 0.88 (0.85, 0.91) |

*, hazard ratio (95% confidence interval); GSI, gallstone-related infection

Supplement 3. Cumulative all-cause mortality rates of myocardial infarction (MI) and cerebral infarction (CI) according to gallstone-related infection (GSI) and cholecystectomy. (A, B) myocardial infarction, A; gallstone-related infection (GSI), B: non-GSI. (C, D) cerebral infarction, C: GSI, D: non-GSI. op, cholecystectomy

**
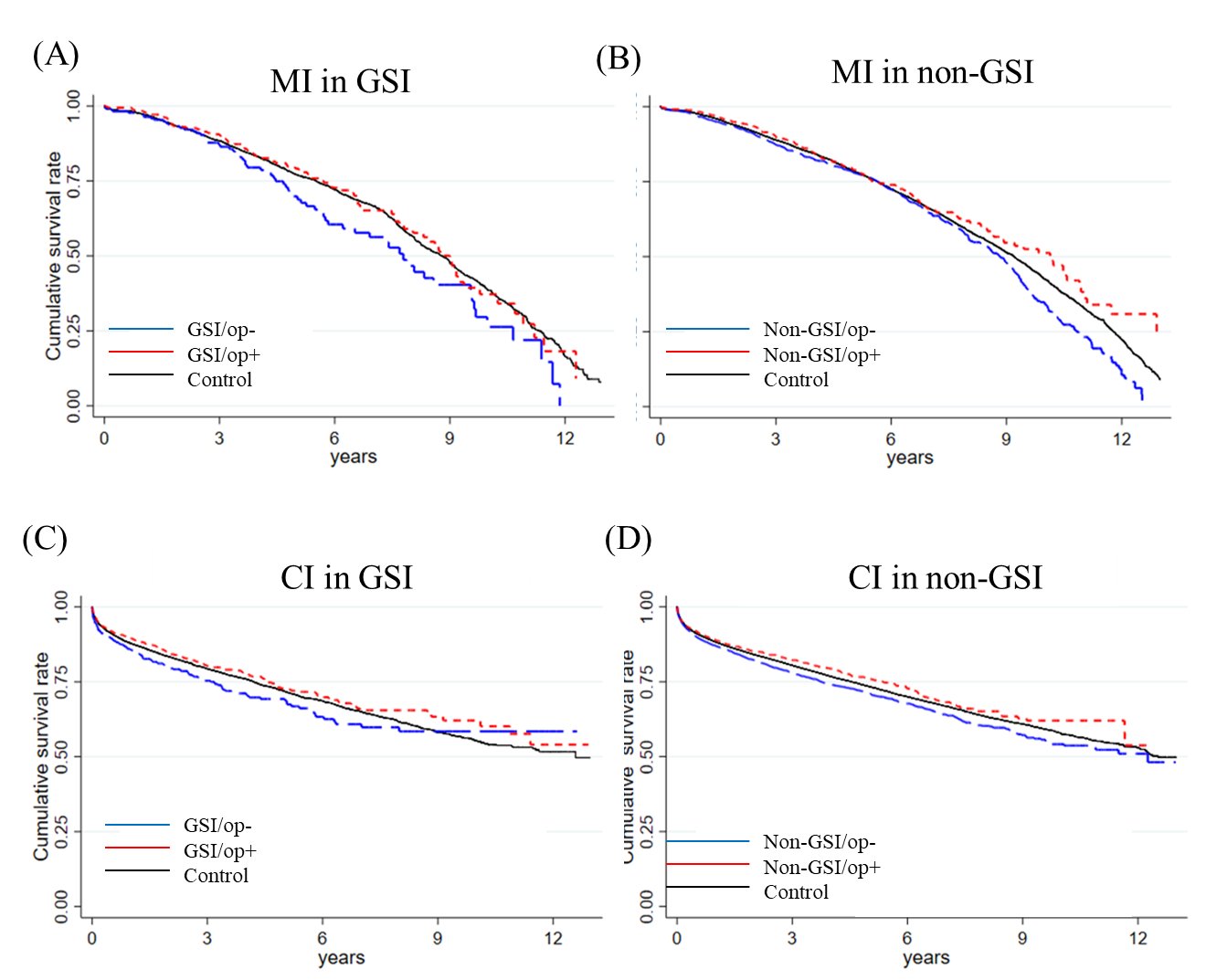
**
